# Culture, inequality, and health: evidence from the MIDUS and MIDJA comparison

**DOI:** 10.1007/s40167-015-0025-0

**Published:** 2015-01-21

**Authors:** Carol D. Ryff, Yuri Miyamoto, Jennifer Morozink Boylan, Christopher L. Coe, Mayumi Karasawa, Norito Kawakami, Chiemi Kan, Gayle D. Love, Cynthia Levine, Hazel R. Markus, Jiyoung Park, Shinobu Kitayama

**Affiliations:** 1 Institute on Aging, University of Wisconsin-Madison, 2245 MSC, 1300 University Ave., Madison, WI 53706 USA; 2 Department of Psychology, University of Wisconsin-Madison, 418 Psych Bldg. 1202 W. Johnson St., Madison, WI 53706 USA; 3 Department of Psychiatry, University of Pittsburgh, 3811 O’Hara St., Pittsburgh, PA 15213 USA; 4 Department of Psychology, University of Wisconsin-Madison, 153 Harlow Primate Lab, 22 N. Charter St., Madison, WI 53715 USA; 5 Department of Communications, Tokyo Woman’s Christian University, 2-6-1 Zempukuji, Suginami-ku, Tokyo 167-8585 Japan; 6 Department of Mental Health, Graduate School of Medicine, University of Tokyo, 7-3-1 Hongo, Kunkyo-ku, Tokyo 113-0033 Japan; 7 Northwestern University Foundations of Health Research Center, 1801 Maple Ave., Ste. 2450, Evanston, IL 60201 USA; 8 Department of Psychology, Stanford University, Bldg. 420, Standford, CA 94305 USA; 9 University of California-San Francisco, 3333 Calif. St., Laurel Heights, Box 0848, San Francisco, CA 94143 USA; 10 Department of Psychology, University of Michigan, 6118 Institute for Social Research, 426 Thompson Street, Ann Arbor, MI 48106 USA

**Keywords:** Culture, Inequality, Health, MIDUS, MIDJA

## Abstract

This article seeks to forge scientific connections between three overarching themes (culture, inequality, health). Although the influence of cultural context on human experience has gained notable research prominence, it has rarely embraced another large arena of science focused on the influence social hierarchies have on how well and how long people live. That literature is increasingly focused psychosocial factors, working interactively with biological and brain-based mechanisms, to account for why those with low socioeconomic standing have poorer health. Our central question is whether and how these processes might vary by cultural context. We draw on emerging findings from two parallel studies, Midlife in the U.S. and Midlife in Japan, to illustrate the cultural specificity evident in how psychosocial and neurobiological factors are linked with each other as well as how position in social hierarchies matters for psychological experience and biology. We conclude with suggestions for future multidisciplinary research seeking to understand how social hierarchies matter for people’s health, albeit in ways that may possibly differ across cultural contexts.

Cultural variation in behavior arises from difference in ideas, interactions, and institutions that characterize various social contexts and reflects differences in how individual brains and bodies attune to these environments. Through their thoughts, feelings, and actions people help to perpetuate the cultures of which they are a part (Markus and Kitayama 2010). The culture variation that has been the focus of most research and is the focus here is between the European American cultural contexts and those of Japan. Of course, variation in behavior is also a function of many other cultures with which people interact, including those of region, race/ethnicity, social class, religion, gender, and generational status. In this article, we bring a targeted focus to cultural variation between two contexts (Japan and the U.S.) and how it matters for inequalities in health linked to socioeconomic status.

Status hierarchies exist in all societies across all cultural contexts and are prominent in most animal species. Increasingly, status hierarchies are recognized as important influences on health. In humans, socioeconomic standing has been shown to influence how long and how well people live (i.e., years without chronic conditions and disabilities) (Adler and Rehkopf 2008; Marmot 2005). Despite these realities, the role of cultural context in understanding how status hierarchies matter for health is not well understood. That limitation is the overarching issue motivating this article. We review evidence from both U.S. and Japanese research, giving emphasis to ways in which the cultural context shapes the nature of the health inequalities observed. How psychosocial and biological factors combine to reveal why those at lower levels of socioeconomic hierarchies often have poorer health is an overarching theme.

Findings from the U.S. are based on the Midlife in the U.S. (MIDUS) investigation, and findings from Japan are based on the Midlife in Japan (MIDJA) study. MIDUS was initiated in 1995 with a national probability sample of over 7,000 U.S. adults aged 25–74. MIDJA was initiated in 2008 (during the 1st longitudinal follow-up of MIDUS) with a probability sample of over 1,000 adults aged 30–79 from the city of Tokyo. Both studies collected a wide array of demographic, psychological and social data. Comprehensive biological measures were also obtained on subsamples of respondents in both countries (U.S. biological subsample = 1,255; Japanese biological subsample = 382) who visited health clinics. The U.S. study included a neuroscience subsample (n = 332) as well, for which there was no equivalent in Japan. Overall, the MIDUS and MIDJA studies are notable in cultural research because they are based on probability samples, thus enriching the socioeconomic heterogeneity of respondents, including wide age diversity, as well as the parallel data collection that was carried out across multiple biopsychosocial domains.

The material that follows is organized into three sections. The first section distills evidence from MIDUS that clarifies how psychosocial factors contribute to health inequalities in the U.S. Some studies identify psychosocial factors that exacerbate the adverse health effects of low status positions, while others point to buffering influences that protect disadvantaged individuals from succumbing to poorer health. The second section highlights emerging findings from MIDJA on socioeconomic gradients in health in the Japanese context. Emphasis is given to how these patterns are distinct from what has been observed in the U.S. Although social inequalities exist in both contexts, there are important differences in how they are manifest. These variants underscore the critical role of culture in shaping, via complex biopsychosocial pathways, the impact of socioeconomic inequality on people’s lives and their health. The third section details promising directions for future inquiries situated at the interface of culture, inequality, and health, including possible socioeconomic differences in health behaviors and practices in the two countries. The promise of brain-based studies to explicate mechanisms underlying cultural similarities and differences in health inequalities is noted as a priority going forward.

## Social inequalities in health: evidence from MIDUS

MIDUS was launched in 1995 with a national sample of over 7,000 U.S. adults aged 25–74 (Brim et al. 2004). Its primary objective was to investigate the role of psychological and social factors in understanding how people age across the decades of adult life. Approximately 10 years later, the National Institute on Aging funded a longitudinal follow-up, which included substantial extensions of the scientific content to include in-depth cognitive, biological, and neuroscience assessments. Today, the enterprise has grown to include comprehensive data collection on nearly 12,000 U.S. adults who are being followed longitudinally. More than 600 publications from MIDUS have appeared in more than 180 scientific journals (see www.midus.wisc.edu). A key theme, among many active areas of inquiry, is health inequalities—how those at the lower end of educational, economic, or occupational status hierarchies have poorer health relative to their more advantaged counterparts, with emphasis on explicating intervening processes. A related theme has been resilience in the face of inequality; that is, how some are able to maintain good health despite having low social status.

An early analysis by Lachman and Weaver (1998) showed that individuals with lower income had worse self-reported health, more depressive symptoms, and lower levels of life satisfaction, when compared to those with higher levels of income. However, showing the protective influence of psychological factors, the authors further reported that the links between income and health were moderated by respondents’ sense of control (mastery). At low levels of mastery, the usual economic gradients in health and well-being were evident. However, at high levels of mastery, the economic gradients disappeared, such that disadvantaged individuals looked comparable to their high income counterparts on measures of health, depressive symptoms, and life satisfaction. Subsequent work by O’Brien (2012) used the MIDUS longitudinal data to investigate whether control beliefs, social support, and social strain moderated the links between educational status and changes in health *over time*. Among those low in educational status, low levels of social strain predicted fewer increments in functional limitations. Alternatively, among those high in educational status, higher level of control predicted fewer increments in depressive symptoms across time. Across all educational groups, those reporting high control and low social strain at baseline showed fewer increments in chronic conditions approximately a decade later. More recently, control beliefs assessed at baseline were found to predict lower risk of mortality 9–10 years later, a relationship that was strongest among those with low educational standing (Turiano et al. 2014). Psychological factors were thus clearly relevant for understanding dynamic changes in health, although how they mattered depended on one’s position in the educational hierarchy.

Thanks to the diverse biological measures collected at MIDUS II on a subsample of respondents, it has been possible to link educational standing to differing factors known to be part of causal pathways to various disease outcomes. Interleukin-6 (IL-6), for example, is a physiological marker of pro-inflammatory activity, which can be readily measured in blood, and is involved in the etiology of numerous diseases (e.g., cardiovascular, Type 2 diabetes, certain cancers, autoimmune conditions such as rheumatoid arthritis, and within the brain, the pathobiology of Alzheimer’s). Morozink et al. (2010) replicated prior findings showing that those with lower levels of education had higher levels of IL-6. However, they further showed that this effect was moderated by various aspects of psychological well-being, such as purpose in life, environmental mastery, positive relations with others, self-acceptance, and positive affect. Interaction patterns revealed that at low levels of well-being, the usual educational gradient in IL-6 was evident; however, at high levels of reported well-being, the educational gradient in levels of IL-6 was absent or notably reduced. These patterns held after adjusting for other sociodemographic factors, medication use and a number of health conditions. In sum, possessing higher levels of multiple aspects of well-being was protective against having elevated IL-6 among those with low levels of educational attainment.

MIDUS has examined negative psychological factors as well, such as anger. Styles of how individuals deal with anger (expressing anger outwardly, holding anger in, controlling anger) have been delineated. Some aspects of anger may be fueled by injustices and blocked goals that characterize life at the bottom of socioeconomic hierarchies, suggesting that anger expression may be a pathway that accentuates health risks among those who are educationally disadvantaged. Boylan and Ryff (2013) tested this idea with the individuals in MIDUS who participated in the biomarker project so as to focus on two pro-inflammatory markers (IL-6, fibrinogen) in the blood stream. As predicted, among those with a high school education or less, higher levels of expressing anger (anger out) were associated with elevated levels of IL-6 as well as the clotting factor fibrinogen. Fibrinogen has been included in many epidemiological studies, as it is associated with cardiovascular disease; high fibrinogen levels increase the viscosity of the blood. Findings also showed that anger control was associated with lower IL-6 among educationally disadvantaged women, but not men. These patterns were evident after adjusting for demographic factors, medication use, and a number of disease conditions. Thus, anger expression was shown to exacerbate the link between low educational standing and two inflammatory markers, while high anger control was found to attenuate risk for elevated IL-6 among less educated women.

Resilience in the face of social inequality has also been linked with biological outcomes. Miller et al. (2011) used MIDUS to investigate associations between childhood poverty and the prevalence of metabolic syndrome in adulthood. Metabolic syndrome is comprised of multiple risk factors for poor health (high blood pressure, impaired glucose control, central adiposity measured by waist circumference, and lipid dysregulation). Collectively, these factors increase the risk for cardiovascular disease and Type 2 diabetes, two illnesses strongly reflective of SES disparities in health in the U.S. Using parental educational as an indicator of childhood disadvantage, the findings first showed that respondents with less educated parents were at higher risk for metabolic syndrome (assessed both categorically and in terms of numbers of metabolic syndrome components) in midlife compared to respondents with better educated parents. However, this educational gradient was moderated by whether respondents reported having a nurturing mother in childhood. Among those with low maternal nurturance, the components of metabolic syndrome were highest among adults with less educated parents. However, among those reporting that they had a nurturing mother, the educational gradient in metabolic syndrome was no longer evident. Thus, high maternal nurturance in childhood offered protection against risk of metabolic syndrome in adult life for those who came from socioeconomically disadvantaged family backgrounds.

Other investigators have used MIDUS to investigate the relevance of additional psychological factors, namely, personality traits, in understanding educational gradients in health outcomes and health behaviors. That is, position in social hierarchies brings social structural influences into the health arena, whereas personality traits bring individual different factors into the prediction of health outcomes. Chapman et al. (2010) argued that socioeconomic factors and personality traits should be considered together in examining antecedents of mortality. Specifically, they found that 20 % of the SES gradient in mortality was explained by personality traits, such as neuroticism, extraversion, or conscientiousness. Neuroticism positively influences mortality risk, whereas extraversion and conscientiousness negatively influence mortality risk. Alternatively, only 8 % of the personality risk accounting for mortality was explained using SES factors. Similarly, Chapman et al. (2009) called for an integrated perspective on factors that predict smoking behavior. Low educational standing is a known risk factor for smoking, but growing evidence implicates personality traits as well—conscientiousness has been shown to reduce risk of smoking, whereas neuroticism and openness increase risk of smoking. Their analyses documented that both educational status and personality traits were relevant (after adjusting for numerous confounds) in accounting for who smokes. Such results underscore the importance of considering both individual difference factors (traits) as well as educational standing in formulating effective prevention or intervention programs to reduce the prevalence of smoking in the U. S. How such findings may be relevant for understanding smoking patterns in Japan is examined in the section on future research directions.

Other publications from MIDUS not reviewed here add to the growing evidence on social inequalities in health (see www.midus.wisc.edu). There is also an emerging international literature on this topic (Adler and Rehkopf 2008; Kawachi et al. 2010; Matthews and Gallo 2011), although the role of cultural factors in understanding socioeconomic disparities in health remains largely unexplored. MIDUS showcases the importance of psychosocial factors in exacerbating, or mitigating the health risks associated with SES hierarchies, and in so doing, offers a useful vantage point for considering cultural influences. Research on cultural psychology (Kitayama and Markus 2000; Markus and Kitayama 1991) further underscores the importance of possible cultural variants in which psychological or social factors moderate or mediate SES influences on health. Cultural factors may also be relevant in identifying which psychosocial factors are most likely to “get under the skin”—that is, are linked with the dysregulation of physiological systems. Further, health behaviors are known to vary by one’s position in the social hierarchy, but whether such findings generalize across cultural contexts is not well understood. Emerging findings from MIDJA, based on middle-aged and older adults in Japan, are beginning to address these questions.

## Social inequalities in health: evidence from MIDJA

MIDJA was launched in 2008 with a probability sample of over 1,000 adults (aged 30–79) from the Tokyo metropolitan area in Japan. Survey data were collected to parallel the sociodemographic, psychosocial and self-reported health data from MIDUS. In addition, biomarker data were collected on a subsample of respondents to parallel a subset of the biological assessments obtained from the MIDUS biomarker sample. Prior to launching data collection from the representative sample from Tokyo, pilot data were collected on about 500 adults from a convenience sample of Japanese adults. Publications from both the pilot sample and the main MIDJA sample are described below.

An initial publication comparing MIDJA and MIDUS findings focused on constructs of independence and interdependence as culturally unique predictors of health. Using the MIDJA pilot sample in combination with the MIDUS baseline sample, Kitayama et al. (2010) investigated two hypotheses to test the strength of the linkage between culture, psychological well-being and health. The first hypothesis was that people are motivated to achieve culturally sanctioned goals relevant in their societies—namely, independence (personal control) is the important goal in the U.S., whereas interdependence (relational harmony) is the mandated goal in Japan. Specifically, it was predicted that people’s reported level of perceived constraints (i.e., not being able to do what they wanted to do), would be most strongly influenced by compromised personal control in the U.S., whereas in Japan, it would be more strongly predicted by strained social relationships. The empirical findings supported these predictions.

The second hypothesis was that reports of health and well-being would be more strongly predicted by independence in the U.S., but by interdependence in Japan. Using a composite index of health and well-being, the obtained findings showed that personal control was the stronger predictor of the composite health outcome in the U.S., whereas relational strain was the stronger predictor in Japan. These analyses controlled for age, gender, educational status, and a number of personality traits. Not examined, but a useful query for social inequalities research would involve investigating whether independence is the primary mediator of class/health linkages in the U.S., whereas interdependence may be the key mediator of such linkages in Japan.

Miyamoto and Ryff (2011) used the MIDJA pilot sample along with the MIDUS baseline sample to investigate cultural differences in emotional styles and their implications for health. Extending prior cross-cultural research, they found that Japanese adults were more likely to report experiencing both positive and negative emotions with moderate frequency, something known as a “dialectical emotional style,” which underscored the middle way of emotional experience in East Asian cultural contexts. U.S. adults, in contrast, were more likely to experience a preponderance of positive emotion and a paucity of negative emotion. These differing emotional profiles were analyzed along with self-reported health symptoms to test the hypothesis that a dialectical emotional style would predict better health in Japan, but not in the U.S. The empirical findings supported this hypothesis; thereby documenting that Japanese adults were more likely to experience a balance of positive and negative emotions compared to U.S. adults, and further that this balanced emotional style was linked with fewer health symptoms for Japanese compared to U.S. adults. The investigation did not consider variation by socioeconomic standing, although a relevant question is whether dialectical emotion styles mediate or moderate the impact of SES on health in Japan, whereas non-dialectical emotion styles may serve to mediate or moderate SES-related health effects in the U.S.

Using only the MIDUS data, Fujiwara and Kawachi (2008) linked the concept of social capital, construed as a kind of interdependence (given its emphasis on trust, sense of belonging, and mutual aid) to the likelihood of developing major depression over time. Their analysis supported the prediction that U.S. adults with higher levels of social capital were less likely to develop major depression over time, after taking sociodemographic factors, physical health status and the trait of extraversion into account. This kind of inquiry could be enriched by consideration of cultural influences; that is, social capital may be especially important in protecting against mental health impairments in interdependent cultures. Consistent with this formulation, Park et al. (2013a, b) used the MIDUS and MIDJA data to show that the link between social support (as an index of interdependence) and self-reported physical health was stronger for Japanese adults, especially when they were under high life stress (i.e., in a support-justifying situation), compared to Americans.

Building on the findings in the U.S. highlighting the interplay of SES, anger, and health, Park et al. (2013a, b) brought culture into the theoretical framework to examine cultural specificity in the association between social status and anger expression. They drew on U.S. findings showing that those with lower social status report more anger, presumably related to frustrations associated with unequal access to resources and opportunities as well as increased stress exposure among the socioeconomically disadvantaged. Anger in Japan, in contrast, is more culturally restricted, given the concerns about its potential to disrupt social harmony and relationships. Japanese culture thus places a greater emphasis on the suppression and control of these types of negative emotions. However, not everyone is expected to suppress anger within Japanese society—those with high social status are given cultural license or authorization to express anger. Consistent with this formulation, a comparison of MIDJA and MIDUS II respondents showed that Americans with lower social standing (measured both objectively and subjectively) expressed more anger. Further, this relationship between status and anger was mediated by self-reported frustration. In contrast, and in keeping with the assumption that higher social standing affords a privilege to display anger in Japan, adults in MIDJA with higher social status were found to express more anger. This relationship between status and anger expression was mediated by decision-making authority. It was also found that anger expression was explained by subjective status among Americans, but by objective social status (i.e., education, occupational status) among Japanese adults. This distinction was elaborated in the study below.

Hierarchies can be defined objectively, by educational attainment or income, as well as subjectively, by people’s perception of their standing vis-à-vis others in their community. Curhan et al. (2014) used MIDJA and MIDUS to examine how these two perspectives on social hierarchy predicted psychological well-being. Although both objective and subjective hierarchical assessments have been linked with psychological well-being in the U.S., no prior research had examined such linkages from a cultural perspective on how social status is understood and conferred. Drawing on the independent model of the self, which is prominent in the U.S., the hypothesis was that subjective perceptions of social status would more strongly influence well-being because individuals’ perceptions are seen as primary determinants of thoughts and feelings. In contrast, according to the interdependent model of the self that is typical and culturally appropriate in Japan, people are seen as fundamentally connected to others. In this context, personal thoughts and feelings are secondary to social approval, including how one is seen in the eyes of others. In such contexts, objective benchmarks of social standing, which signal community respect, are likely to be more influential in predicting reported well-being. As hypothesized, subjectively perceived social status was more strongly predictive of life satisfaction, positive affect, sense of purpose, and self-acceptance in the U.S. compared to Japan. Alternatively, objective social status (i.e., educational attainment) was more strongly predictive of life satisfaction, positive relations with others, and self-acceptance in Japan than in the U.S. These findings underscored the importance of considering objective and subjective indicators of position in social hierarchies when studying social inequalities in health across cultures.

Psychological resources, such as one’s sense of control, self-esteem, or optimism, are usefully examined as mediators of the links between position in social hierarchies and health. Kan et al. (2014) investigated these questions by comparing data from MIDJA and MIDUS. Their investigation incorporated both objective (i.e., educational attainment) and subjective assessments of social status, and linked them to self-rated health and number of chronic illness conditions. Given the foregoing findings, the hypothesis was that self-esteem and sense of control would more strongly mediate the relationships between social status and health in the U.S. than in Japan. This prediction was supported for self-esteem, which was found to mediate the links between objective and subjective status and health outcomes in the U.S., but few parallel effects were evident in Japan. Alternatively, sense of personal control was found to mediate both types of status and health outcomes in Japan as well as the U.S. Optimism also significantly mediated most associations of social status and health in both countries, but only among females. Neuroticism, a trait that may be accentuated by the absence of psychological resources, was also found to mediate associations between social status and health in both countries, except for the predictions of chronic conditions among Japanese women. These findings thus pointed to similarities as well as differences across cultures in how psychological factors influence pathways connecting social status to health. That self-esteem was a mediator in the U.S., but not in Japan may reflect the cultural emphasis on self-criticism in Japan. We note that the mediators examined in this study reflected a largely Western conception of psychological resources. Future research might therefore usefully examine whether factors such as social harmony or social obligation have greater prominence as mediators in Japan.

Bringing biological mechanisms into cultural studies of health offers useful insights into which psychological factors are implicated in linkages between mind and body. An important theme in cultural studies has been that negative emotions are construed as more problematic in Western contexts, and indeed, have been found to be predictors of the risk for cardiovascular disease (Kubzansky and Kawachi 2000), cancer (Penninx et al. 1998) and mortality (Pinquart and Duberstein 2010). Biological pathways linking these negative emotions to disease outcomes are beginning to be explicated. One such pathway involves an exacerbation of pro-inflammatory processes. Cultural studies are critical to test whether these linkages are universal across countries as well as across different ethnic and racial groups. The growing literature on how emotions are differently construed and accompanied by differing normative expectations suggests that cultural specificity may be evident. Building on Miyamoto and Ryff (2011), which characterized negative emotion in Western contexts as something to be avoided, whereas in Asian contexts, negative emotions are accepted and recognized as necessary for mature adult development, Miyamoto et al. (2013) tested the hypothesis was that negative emotion would predict elevated levels of the pro-inflammatory cytokine IL-6 in the U.S., but not in Japan. Data from the MIDUS and MIDJA biomarker samples confirmed this prediction. Respondents indicated how often in the past 30 days they had felt a number of negative emotions (e.g., sad, hopeless, worthless, nervous, restless, that everything was an effort). After adjusting for sociodemographic factors, personality traits, positive emotions, health behaviors, and chronic conditions, the findings showed that a person’s cultural background moderated the experience of negative emotion and IL-6. Only in the U.S. did higher levels of negation emotion predict elevated IL-6 levels. Thus, the influence of cultural context must be considered when linking negative emotion to pro-inflammatory markers. Related research (e.g., Pressman et al. 2013), however, has shown that both positive and negative emotions are linked to self-reported health across cultural contexts, suggesting that self-rated health may be colored by one’s emotional state. When objective markers (such as IL-6) are employed, however, our findings did not support a strong negative emotion to health link in Japan. Further inquiry is needed to clarify whether negative emotions are less physiologically costly in Japan perhaps because they are construed as acceptable, and indeed, necessary components of normal adult life. Longitudinal inquiries are also needed to sharpen the understanding of causal relations between emotions and biological risk factors.

More recent work (Kitayama et al. 2015) extended the analysis above with a specific focus on anger expression. In this MIDJA/MIDUS investigation, culture was hypothesized to moderate the link between anger expression and a composite index of biological health risk, which included two pro-inflammatory markers (IL-6 and CRP) and two indices of cardiovascular dysfunction (high systolic blood pressure and a poor cholesterol profile). Because prior findings (Park et al. 2013a, b) showed that anger was differentially experienced and expressed in each country (i.e., more common among low status adults in the U.S., but high status adults in Japan), we expected that anger might be associated with increased biological risk in the U.S., but the opposite effect in Japan, namely decreased biological risk. After adjusting for demographic factors, health status, and health behaviors, cultural context was found to moderate, as predicted, the link between anger expression and the indices of biological risk. Further, the pattern held even after adjusting for differences in social status, measured both objectively and subjectively. Thus, the cultural moderation was largely independent of social status. Other factors, as yet to be identified, may account for why high anger expression is associated with biological risk among U.S. adults, but can be health-enhancing among Japanese adults.

To summarize, MIDJA and MIDUS have advanced understanding of how culture moderates the influence of psychosocial factors on health and well-being. Not all of these analyses have focused on inequality, but the extant work does demonstrate that there are cultural differences in how much hierarchies (measured objectively and subjectively) matter for health. Further, the studies show that position in the social hierarchy can influence the expression of particular emotions, such as anger, in ways that are culturally distinct. Further research is needed to replicate and extend these lines of inquiry, including to other countries and cultures. The final section below addresses useful future directions for advancing knowledge on social inequalities in health in ways that are informed by a culturally sensitive perspective.

## Promising directions for future inquiry

Although extensive research, including international studies, has been generated on health inequalities, how cultural context influences the ways in which socioeconomic hierarchies matter for health has received limited attention. This omission is notable, given that culture likely pervades many core questions about whether psychosocial factors act as protective or exacerbating influences among disadvantaged members of society. In what follows, two lines of research showing promise for future studies are outlined. The first focuses on the need to bring health behaviors into research on culture, inequality, and health, and the second addresses what neuroscience might contribute to this integrative research agenda.

### Culture, inequality and health behaviors

Health behaviors are strongly implicated in understanding social inequalities in morbidity and mortality. Those at the lower end of the socioeconomic hierarchy, especially in the U.S., engage in more compromising behaviors, such as overeating, sometimes in response to stress (Tsenkova et al. 2013), smoking (Chapman et al. 2009), less exercise (Grzywacz and Marks 2001), and have poorer sleep habits (Mezick et al. 2008; Van Cauter and Spiegel 1999). An important issue is whether there are cultural differences in the patterning of these health behaviors by social status, and relatedly, whether there are cultural differences in psychosocial and emotional factors that may be antecedents to unhealthy behaviors. An interesting example is smoking, a health behavior of known risk for cancer and heart disease that is disproportionately evident among the less educated segments of the U.S. population (Warner 2000).

MIDUS and MIDJA offer contrasting perspectives on educational status and smoking (Ryff 2014a). For example, in the U.S., better educated adults are less likely to have ever smoked compared to less educated adults. In contrast, in Japan, adults with more education are more likely to have ever smoked compared to less educated adults. Distinctions are also evident when examining who has quit smoking as a function of educational status: among those who ever smoked, about 80 % of college educated U.S. adults report no longer smoking, compared to 50 % of college educated Japanese adults. Further, rates of smoking cessation are lower among high-school or less educated adults, but again, there are notable differences by country: over 60 % of less educated U.S. adults who ever smoked are not currently smoking, compared to over 40 % in Japan. Gender also exerts a prominent influence on smoking behavior: those smoking the greatest number of cigarettes per day in Japan are college educated men, whereas Japanese women with a college education smoke the least. In the U.S., those smoking the greatest number of cigarettes are high school educated men, whereas college educated women, as in Japan, smoke the least.

Largely unexplored and therefore notably ripe for scientific inquiry are studies of the psychological and social factors that may increase or decrease the likelihood of smoking, and whether such influences vary by cultural context. Negative emotions, for example, may fuel smoking to a greater extent in the U.S. than in Japan. It is also possible that susceptibility to smoking depends on culturally specific meanings of smoking. If smoking is recognized largely as a health risk, educational attainment may lead to reduced smoking, as in the U.S. But smoking may also be a means to express masculinity and show social or personal prowess. If so, men, especially those who have access to power, may smoke more—a pattern that may be applicable in Japan. More research is needed to understand how these cultural and contextual (e.g., workplace) factors influence smoking as well as smoking cessation. Findings from the previously described U.S. research (Chapman et al. 2009) suggest that individual difference variables, such as personality traits (neuroticism, openness), may amplify tendencies to smoke, whereas others (conscientiousness) may reduce risk of smoking. Such patterns may not generalize to Japan, if smoking has different cultural meanings in that context and is possibly a behavior indicating strength or masculinity.

Cultural influences on diet and nutrition are a current focus in MIDUS and MIDJA (Levine et al. 2014, unpublished manuscript). Both studies collected data on healthy eating, defined in terms of consumption of white to red meat, non-meat protein, and fruit and vegetables. A core idea guiding the analysis was that independence, which captures the sense of agency and individualism prominent in the U.S., would better predict healthy eating in the U.S. Alternatively, interdependence, which captures the fundamental other-connectedness and importance of social relationships in Japan, would better predict healthy eating in Japan. These predictions were supported. In addition, findings showed that the obtained linkages were mediated by culture-specific factors. Sense of control served as the mediator of the link between independence and healthy eating in the U.S., whereas spousal support served as the mediator of the link between interdependence and healthy eating in Japan. Going forward, it would be useful to bring social status perspectives into assessments of healthy eating across cultures. In the U.S., accessibility of healthy food may be more closely linked with socioeconomic status (Walker et al. 2010), whereas in Japan, healthy food may be more readily accessible to people from diverse socioeconomic backgrounds.

Another promising line of inquiry pertains to drinking. Useful questions are whether patterns of drinking (age of first drinking, amount of alcohol consumption) differ between Japanese and U.S. adults, and further whether there are social gradients in such profiles in both countries. It would also be useful to incorporate constructs of independence and interdependence to investigate the cultural moderation of psychosocial factors that predict alcohol consumption. Finally, the use of alternative therapies may be fruitful to pursue in comparing Western and East Asian cultural contexts. MIDUS assessed therapies such as acupuncture, biofeedback, energy healing, herbal therapy, massage therapy, medication techniques, and spiritual practices. McMahan and Lutz (2004) used these data to examine what sociodemographic factors predicted the use of alternative and complementary medicine among older adults (defined as ages 65–74). Educational standing was a significant positive predictor, as was higher income and being female. Because many alternative therapies have their origins in Eastern cultures, it would be informative to examine the prevalence of these practices in Japan as well, along with whether education, income, or gender matter. And again, incorporating constructs of independence and interdependence into the formulations would strengthen understanding of whether these practices are contoured by culturally-specific formulations of the self.

### Culture, inequality, and the brain

In recent years, there have been calls for increased attention to the neurobiological pathways through which socioeconomic position is linked to health. Gianaros and Manuck (2010) considered neuropharmacological, molecular genetic, and neuroimaging studies that hold promise for enhancing understanding of how the brain is implicated in SES and health linkages. Using both human and animal models, they underscored the scope of prior work connecting chronic stressors and mood systems to the brain, both of which are relevant for understanding the health consequences of social inequalities. Consistent with results highlighted above, they also emphasized the importance of attending to subjective indicators of socioeconomic position. Lower self-rated social status was linked, for example, to reduced gray matter volume in the perigenual area of the anterior cingulate cortex (Gianaros et al. 2007), while perceiving that one’s parents had low social standing was linked with greater amygdala reactivity to threatening facial expressions (Gianaros et al. 2008).

Alternatively, Hackman et al. (2010) approached the topic of socioeconomic status and the brain from an early life perspective, giving emphasis to the role of prenatal factors, parental care and cognitive stimulation, all possibly shaped by socioeconomic standing, in a child’s neurocognitive development. Hillman et al. (2008) further emphasize the importance of aerobic physical activity on cognition and brain function at the molecular, cellular, systems, and behavioral levels. Although not explicitly focused on socioeconomic factors, their review encompasses the life course (childhood, early adulthood, old age) and links lack of physical activity to a major health problem (obesity) known vary by social strata.

Outside the realm of social inequalities, there is growing interest in how brain-based assessments of emotion regulation might be meaningfully linked to physical health. Song et al. (2014), for example, summarized emerging research on mind–body interactions and called for more inquiry on the neural bases through which emotion regulation is linked to physical health. Working with a Chinese sample, they used magnetic resonance imaging (MRI) to show that individuals who had more gray matter (GM) volumes in the amygdala reported not only better emotion regulation (assessed with a stress management inventory), but also better self-rated physical health. Further, they found that GM volume in the amygdala appeared to serve a mediating role in the association between emotion regulation ability and physical health. Given that emotion regulation, as emphasized in preceding sections, may take different forms in Western versus East Asian cultures, future research is needed to examine whether the neural pathways linking emotion regulation to better physical health in this Chinese sample will generalize to Western populations.

Others studies have examined the neural correlates of well-being. Lewis et al. (2014), for example, focused on insular cortex grey matter volume and showed positive links to several aspects of eudaimonic well-being, including purpose in life, personal growth, and positive relations. Another study by Kong et al. (2014) used resting-state functional MRI to show different neural substrates (i.e., right posterior superior temporal gyrus, thalamus-insular connectivity) through which eudaimonic well-being is associated with personality traits. Further neuroimaging research by van Reekum et al. (2007) found that those who were faster to evaluate negative information showed greater activation of the amygdala, but such relationships were moderated by reports of eudaimonic well-being: those with higher well-being were slower to evaluate the negative stimuli and evinced less activation of the amygdala; they also showed increased activation of the anterior cingulate cortex. The overall pattern suggested that people higher in well-being effectively recruit the ventral anterior circulate cortex when confronted with aversive stimuli, which may partially account for their reduced activity in the amygdala and slower speed in evaluating the information.

None of the preceding well-being studies addressed possible linkages to health, although many aspects of eudaimonic well-being, as reviewed by Ryff (2014b), have been shown to predict morbidity, mortality, and biological risk factors. Similarly, concern with cultural context is notably missing in the neuroscience work described above, although MIDUS and MIDJA findings have underscored cultural differences in conceptions of well-being (see Ryff et al. 2014). These differences suggest there may be cultural specificity in which aspects of reported well-being are most strongly linked with grey matter volume, or with activity in the amygdala, or anterior cingulate cortex. Relational harmony may be a stronger neural correlate for Japanese adults than eudaimonic well-being. Such possibilities raise further queries regarding the generalizability of prior U.S. research, which has linked social neuroscience to health (Eisenberger and Cole 2012), or incorporated social support as a moderator of the link between threat-related amygdala reactivity and trait anxiety (Hyde et al. 2011). Undoubtedly, psychological and social experiences are implicated in brain activity in all cultures, but whether there are cultural differences in the nature of such patterns is largely unexplored.

We conclude this section with recent neuroscience findings from MIDUS, which includes a laboratory protocol that obtains brain-based measures of emotional reactivity and recovery on a subsample of U.S. respondents. Using the eyeblink startle reflex (EBR), known to be sensitive to one’s emotional state, Schaefer et al. (2013) examined links between purpose in life (a single aspect of eudaimonic well-being known to predict morbidity and mortality, see Ryff 2014b) and emotional recovery from negative stimuli. The analyses differentiated between initial emotional reactivity (during stimulus presentation) and emotional recovery (occurring after stimulus offset). Greater purpose in life, assessed 2 years prior, was found to predict better recovery from negative stimuli, after controlling for initial reactivity, gender, age, trait affect, and other dimensions of well-being. The findings suggested that one proximal mechanism through which purpose in life may afford protection from negative stressors is via enhanced autonomic regulation after negative emotional provocation.

Another recent MIDUS study Heller et al. (2013) probed brain-based processes that underlie well-being and incorporated assessments of neuroendocrine regulation. The key focus was on the capacity of some individuals to engage the neural circuitry of reward for longer periods; this capacity may constitute part of overall well-being and it may mediate links between well-being and adaptive biological regulation (measured in terms of cortisol output). Participants viewed positive, neutral, and negative images while fMRI data were collected. Those showing sustained activity in the striatum and dorsal lateral prefrontal cortex in response to positive stimuli reported higher well-being. In addition, they showed lower cortisol output, measured four times a day over 4 days. Overall the results pointed to neural mechanisms that possibly underlie the experience of high well-being, with accompanying contributions to adaptive regulation of the hypothalamic–pituitary–adrenal axis.

Finally, because MIDUS is longitudinal, it has been possible to link long-term profiles of psychosocial factors to laboratory-based measures of emotional reactivity and recovery. Lapate et al. (2014) examined how prolonged marital stress, assessed over a 9–10 year period, was linked with reactivity to and recovery from emotional provocation. Respondents watched positive, neutral, and negative pictures while an objective measure of affective state known as supercilii muscle activity was continuously recorded. The findings indicated that marital stress among these middle-aged men and women was associated with short-lived responses to positive pictures, indexed by less persistent decrease in corrugator activity after picture offset. The interpretation provided was that marital stress may impact emotional health over time by compromising brain-based processes involved in short-lived responses to positive stimuli. Further, decreased maintenance of positive affect worked as a mediator of the link between marital strain and depressive symptoms. The article also addressed possible neurobiological mechanisms through which prolonged exposure to a stressful social environment might alter affective responses to positive stimuli, such as reduced engagement of basal ganglia circuitry.

Relevant cultural questions can be raised about all of the neuroscience findings from MIDUS. From a cultural perspective, it is useful to ask whether decreased maintenance of positive affect is equally harmful for health across cultures. Asians are less likely than Americans to try to maintain positive emotions after experiencing a positive event (Miyamoto and Ma 2011), suggesting that they may show short-lived affective responses to positive pictures. Furthermore, decreased maintenance of positive affect may be more weakly associated with stressful social environments or depressive symptoms in Asian cultural contexts, where positive emotions are less socially valued (Bastian et al. 2014).

Similarly, when culture is brought into the interplay of emotion and the brain, a different aspect of emotion regulation, namely, emotional suppression may be important. Murata et al. (2013) found differences in European Americans and East Asians in electrocortical responses during a laboratory-based emotion suppression task. Because Asians are culturally trained to control and suppress their emotional expressions, the key hypothesis was that they would show greater decrease in the parietal late positive potential (LPP, an aspect of event related brain potential, ERP, involved in emotional processing) in a suppression task compared to an attend task compared to European American respondents. Findings supported this prediction, thereby documenting the important role of culture in understanding brain-based assessments of emotional suppression. Thus, whatever may constitute the neural indicators of interest, the formulation of what defines effective emotional regulation may well require specificity across cultural contexts.

Regarding longitudinal relational experiences, a relevant cultural question is what kinds of psychosocial experience over time might be implicated in accounting for changes laboratory-based assessments of emotional reactivity and recovery among Asians. Western indicators of marital strain focus on whether one’s spouse is a source of demands, criticism, tension/arguments, annoyance, and feelings of being let down. Whether these are relevant indicators of marital stress in the Eastern context is unclear. Because negative emotion is construed to be more normative in such settings, such measures of tension and annoyance may simply be less predictive of neural signals, in the same way that negative emotion was shown above to not be predictive of elevated IL-6 in Japan, even though such effects were evident in the U.S. (Miyamoto et al. 2013). What may be more informative in interdependent cultures are relational components of well-being (e.g., sympathy, attunement to others). Unfortunately, MIDJA has not included a neuroscience project to date, thus precluding investigation of these questions.

Returning to the overarching question of health inequalities, it is important to consider how linkages of psychosocial and emotional factors with brain-based processes might incorporate social status hierarchies as part of the overall formulation. Socioeconomically disadvantaged individuals in Western studies are known to have greater exposures to stress as well as poorer coping resources (Matthews and Gallo 2011). Findings from MIDUS have clarified how other psychological factors, may protect against (e.g., well-being), or amplify (e.g., anger) the adverse health impacts of low socioeconomic standings. Findings from MIDJA have clarified that status matters in Japan as well, but in ways that differ from the U.S. (e.g., anger is sanctioned among those high in status and is not linked to biological risk factors). Much remains to be done to explicate the neural underpinnings of these linkages among status, psychosocial factors, and biology. To carry this agenda forward, it will be essential to bring socioeconomically disadvantaged individuals into neuroscience laboratories across cultural contexts. Thus, sampling heterogeneity must pertain not only to diverse cultural contexts, but also to variability related to distinct positions in known social hierarchies.

In conclusion, there are many important future questions for science situated at the interface of culture, inequality, and health. These directions, stimulated by emerging findings from MIDUS and MIDJA, speak to the fundamental role of cultural context in understanding the integration of behavior, psychological experience, biology, neuroscience, and health. Bringing inequality into such expansive inquiries serves as a reminder that in all Eastern and Western cultures, there are layers of meaning and experience tied to differences in social status hierarchies. Understanding how life is construed by those with low compared to high status positions (e.g., Markus et al. 2004) is thus a complex, but worthy pursuit, given that status hierarchies appear to matter for health, via complex psychosocial and neurobiological pathways, across cultural contexts.

## References

[CR1] Adler NE, Rehkopf DH (2008). U.S. Disparities in health: Descriptions, causes, and mechanisms. Annual Review of Public Health.

[CR2] Bastian B, Kuppens P, De Roover K, Diener E (2014). Is valuing positive emotion associated with life satisfaction?. Emotion.

[CR3] Boylan JM, Ryff CD (2013). Varieties of anger and the inverse link between education and inflammation: Toward an integrative framework. Psychosomatic Medicine.

[CR4] Brim OG, Ryff CD, Kessler RC, Brim OG, Ryff CD, Kessler RC (2004). The MIDUS national survey: An overview. How healthy are we?: A national study of well-being at midlife.

[CR5] Chapman B, Fiscella K, Duberstein P, Kawachi I (2009). Education and smoking: Confounding or effect modification by phenotypic personality traits?. Annals of Behavioral Medicine.

[CR6] Chapman BP, Fiscella K, Kawachi I, Duberstein PR (2010). Personality, socioeconomic status, and all-cause mortality in the United States. American Journal of Epidemiology.

[CR8] Curhan KB, Levine CS, Markus HR, Kitayama S, Park J, Karasawa M (2014). Subjective and objective hierarchies and their relations to psychological well-being: A U.S./Japan comparison. Social Psychological and Personality Science.

[CR9] Eisenberger NI, Cole SW (2012). Social neuroscience and health: Neurophysiological mechanisms linking social ties with physical health. Nature Neuroscience.

[CR10] Fujiwara T, Kawachi I (2008). A prospective study of individual-level social capital and major depression in the United States. Journal of Epidemiology and Community Health.

[CR11] Gianaros PJ, Horenstein JA, Cohen S, Matthews KA, Brown SM, Flory JD, Critchley HD, Manuck SB, Hariri AR (2007). Perigenual anterior cingulate morphology covaries with perceived social standing. Social Cognitive and Affective Neuroscience.

[CR12] Gianaros PJ, Horenstein JA, Hariri AR, Sheu LK, Manuck SB, Matthews KA (2008). Potential neural embedding of parental social standing. Social Cognitive and Affective Neuroscience.

[CR13] Gianaros PJ, Manuck SB (2010). Neurobiological pathways linking socioeconomic position and health. Psychosomatic Medicine.

[CR14] Grzywacz JG, Marks NF (2001). Social inequalities and exercise during adulthood: Toward an ecological perspective. Journal of Health and Social Behavior.

[CR15] Hackman DA, Farah MJ, Meaney MJ (2010). Socioeconomic status and the brain: Mechanistic insights from human and animal research. Nature Reviews Neuroscience.

[CR16] Heller AS, van Reekum CM, Schaefer SM, Lapate RC, Radler BT, Ryff CD (2013). Sustained ventral striatal activity predicts eudaimonic well-being and cortisol output. Psychological Science.

[CR17] Hillman CH, Erickson KI, Kramer AF (2008). Be smart, exercise your heart: Exercise effects on brain and cognition. Nature Reviews Neuroscience.

[CR18] Hyde LW, Gorka A, Manuck SB, Hariri AR (2011). Perceived social support moderates the link between threat-related amygdala reactivity and trait anxiety. Neuropsychologia.

[CR19] Kan C, Kawakami N, Karasawa M, Love GD, Coe CL, Miyamoto Y (2014). Psychological resources as mediators of the association between social class and health: Comparative findings from Japan and the USA. International Journal of Behavioral Medicine.

[CR20] Kawachi I, Adler NE, Dow WH (2010). Money, schooling, and health: Mechanisms and causal evidence. Annals of the New York Academy of Sciences.

[CR21] Kitayama S, Karasawa M, Curhan KB, Ryff CD, Markus HR (2010). Independence and interdependence predict health and wellbeing: Divergent patterns in the United States and Japan. Frontiers in Psychology.

[CR22] Kitayama S, Markus HR, Diener E, Suh EM (2000). The pursuit of happiness and the realization of sympathy: Cultural patterns of self, social relations, and well-being. Culture and subjective well-being.

[CR23] Kitayama, S., Park, J., Boylan, J. M., Miyamoto, Y., Levine, C. S., Markus, H. R., Karasawa, M., Coe, C. L., Kawakami, N., Love, G. D., & Ryff, C. D. (2015). Expression of anger and ill health in two cultures: An examination of inflammation and cardiovascular risk. *Psychological Science* (in press). 10.1177/0956797614561268 PMC4323672 25564521

[CR24] Kong F, Liu L, Wang X, Hu S, Song Y, Liu J (2014). Different neural pathways linking personality traits and eudaimonic well-being: A resting-state functional magnetic resonance imaging study. Cognitive, Affective and Behavioral Neuroscience.

[CR25] Kubzansky LD, Kawachi I (2000). Going to the heart of the matter: Do negative emotions cause coronary heart disease?. Journal of Psychosomatic Research.

[CR26] Lachman ME, Weaver SL (1998). The sense of control as a moderator of social class differences in health and well-being. Journal of Personality and Social Psychology.

[CR27] Lapate RC, van Reekum CM, Schaefer SM, Greischar LL, Norris CJ, Bachhuber DR (2014). Prolonged marital stress is associated with short-lived responses to positive stimuli. Psychophysiology.

[CR28] Lewis GJ, Kanai R, Rees G, Bates TC (2014). Neural correlates of the ‘good life’: Eudaimonic well-being is associated with insular cortex volume. Social Cognitive and Affective Neuroscience.

[CR29] Markus HR, Kitayama S (1991). Culture and the self: Implications for cognition, emotion, and motivation. Psychological Review.

[CR30] Markus HR, Kitayama S (2010). Cultures and selves: A cycle of mutual constitution. Perspectives on Psychological Science.

[CR31] Markus, H. R., Ryff, C. D., Curhan, K. B., & Palmersheim, K. A. (2004). In their own words: Well-being at midlife among high school-educated and college-educated adults. In O. G. Brim, C. D. Ryff, & R. C. Kessler (Eds.), *How healthy are we?: A national study of well*-*being at midlife* (pp. 273–319, The John D. and Catherine T. MacArthur foundation series on mental health and development. Studies on successful midlife development). Chicago: The University of Chicago Press.

[CR32] Marmot MG (2005). Status syndrome: How social standing affects our health and longevity.

[CR33] Matthews KA, Gallo LC (2011). Psychological perspectives on pathways linking socioeconomic status and physical health. Annual Review of Psychology.

[CR34] McMahan S, Lutz R (2004). Alternative therapy use among the young-old (ages 65–74): An evaluation of the MIDUS database. Journal of Applied Gerontology.

[CR35] Mezick EJ, Matthews KA, Hall M, Strollo PJJ, Buysse DJ, Kamarck TW (2008). Influence of race and socioeconomic status on sleep: Pittsburgh sleepscore project. Psychosomatic Medicine.

[CR36] Miller GE, Lachman ME, Chen E, Gruenewald TL, Karlamangla AS, Seeman TE (2011). Pathways to resilience: Maternal nurturance as a buffer against the effects of childhood poverty on metabolic syndrome at midlife. Psychological Science.

[CR37] Miyamoto Y, Boylan JM, Coe CL, Curhan KB, Levine CS, Markus HR (2013). Negative emotions predict elevated interleukin-6 in the United States but not in Japan. Brain, Behavior, and Immunity.

[CR38] Miyamoto Y, Ma X (2011). Dampening or savoring positive emotions: A dialectical cultural script guides emotion regulation. Emotion.

[CR39] Miyamoto Y, Ryff CD (2011). Cultural differences in the dialectical and non-dialectical emotional styles and their implications for health. Cognition and Emotion.

[CR40] Morozink JA, Friedman EM, Coe CL, Ryff CD (2010). Socioeconomic and psychosocial predictors of interleukin-6 in the MIDUS national sample. Health Psychology.

[CR41] Murata A, Moser JS, Kitayama S (2013). Culture shapes electrocortical responses during emotion suppression. Social Cognitive and Affective Neuroscience.

[CR42] O’Brien K (2012). Healthy, wealthy, wise? Psychosocial factors influencing the socioeconomic status-health gradient. Journal of Health Psychology.

[CR43] Park J, Kitayama S, Karasawa M, Curhan K, Markus HR, Kawakami N (2013). Clarifying the links between social support and health: Culture, stress, and neuroticism matter. Journal of Health Psychology.

[CR44] Park J, Kitayama S, Markus HR, Coe CL, Miyamoto Y, Karasawa M (2013). Social status and anger expression: The cultural moderation hypothesis. Emotion.

[CR45] Penninx BWJH, Guralnik JM, Havlik RJ, Pahor M, Ferrucci L, Cerhan JR (1998). Chronically depressed mood and cancer risk in older persons. Journal of the National Cancer Institute.

[CR46] Pinquart M, Duberstein PR (2010). Depression and cancer mortality: A meta-analysis. Psychological Medicine.

[CR47] Pressman SD, Gallagher MW, Lopez SJ (2013). Is the emotion-health connection a “first-world problem”?. Psychological Science.

[CR48] Ryff, C. D. (2014a). Bringing culture and neuroscience to research on social inequalities and health. In *2014 International Cultural Neuroscience Consortium Conference* (Vol. Talk). Ann Arbor, MI: University of Michigan, April 4 2014.

[CR49] Ryff CD (2014). Psychological well-being revisited: Advances in the science and practice of eudaimonia. Psychotherapy and Psychosomatics.

[CR50] Ryff, C. D., Love, G. D., Miyamoto, Y., Markus, H. R., Curhan, K. B., & Kitayama, S., et al. (2014). Culture and the promotion of well-being in East and West: Understanding varieties of attunement to the surrounding context. In G. A. Fava, & C. Ruini (Eds.), *Increasing psychological well*-*being in clinical and education settings: Interventions and cultural contexts* (Cross-cultural advancements in positive psychology, Vol. 8). New York: Springer.

[CR51] Schaefer SM, Boylan JM, van Reekum CM, Lapate RC, Norris CJ, Ryff CD (2013). Purpose in life predicts better emotional recovery from negative stimuli. PLoS One.

[CR52] Song Y, Lu H, Hu S, Xu M, Li X, Liu J (2014). Regulating emotion to improve physical health through the amygdala. Social Cognitive and Affective Neuroscience.

[CR53] Tsenkova V, Boylan JM, Ryff C (2013). Stress eating and health: Findings from MIDUS, a national study of U.S. Adults. Appetite.

[CR54] Turiano NA, Chapman BP, Agrigoroaei S, Infurna FJ, Lachman M (2014). Perceived control reduces mortality risk at low, not high, education levels. Health Psychology.

[CR55] Van Cauter E, Spiegel K (1999). Sleep as a mediator of the relationship between socioeconomic status and health: A hypothesis. Annals of the New York Academy of Sciences.

[CR56] van Reekum CM, Urry HL, Johnstone T, Thurow ME, Frye CJ, Jackson CA (2007). Individual differences in amygdala and ventromedial prefrontal cortex activity are associated with evaluation speed and psychological well-being. Journal of Cognitive Neuroscience.

[CR57] Walker RE, Keane CR, Burke JG (2010). Disparities and access to healthy food in the United States: A review of food deserts literature. Health and Place.

[CR58] Warner KE, Smedley BD, Syme LS (2000). The need for, and value of, a multi-level approach to disease prevention: The case of tobacco control. Promoting health: Intervention strategies from social and behavioral research.

